# Primary eccrine porocarcinoma of the thumb with multiple metastases: a case report and review of the literature

**DOI:** 10.1080/23320885.2019.1647108

**Published:** 2019-07-26

**Authors:** Connor McGuire, Zahir Fadel, Osama Samargandi, Jason Williams

**Affiliations:** a Faculty of Medicine, Dalhousie University, Halifax, Nova Scotia, Canada;; b Division of Plastic and Reconstructive Surgery, Department of Surgery, Halifax, Nova Scotia, Canada;; c Division of Plastic and Reconstructive Surgery, Department of Surgery, King Abdulaziz University, Jeddah, Saudi Arabia

**Keywords:** Porocarcinoma, thumb, metastasis

## Abstract

We present a case of primary eccrine porocarcinoma of the thumb in a 56-year-old male who ultimately developed multiple metastases. With so few cases of such lesions and their aggressive nature, accurate diagnosis and prompt surgical management is essential.

## Introduction

Eccrine porocarcinomas are a rare malignant type of sweat gland tumor that were first described by Pinkus and Mehregan in 1963 [1]. The incidence of eccrine porocarcinoma has been estimated at one case per 25,000 skin biopsies, although exact figures do not exist [1,2]. Although porocarcinomas are rare, it is even more uncommon to have a malignant porocarcinoma on the hand. Most cases described are found in females, on the lower extremity or trunk region, and without lymphovascular invasion [3]. While typically not fatal, eccrine porocarcinomas have the ability to be easily misinterpreted as seborrheic warts or even basal or squamous cell carcinomas.

We detail a case of eccrine porocarcinoma of the thumb that ultimately developed multiple metastases, with a subsequent discussion emphasizing the difficulties of initial identification and principles of surgical resection. All procedures followed were in accordance with the ethical standards of the responsible committee on human experimentation (institutional and national) and with the Helsinki Declaration of 1975, as revised in 2008. Informed consent was obtained from the patient included in the study.

## Case report

**Figure 1.**
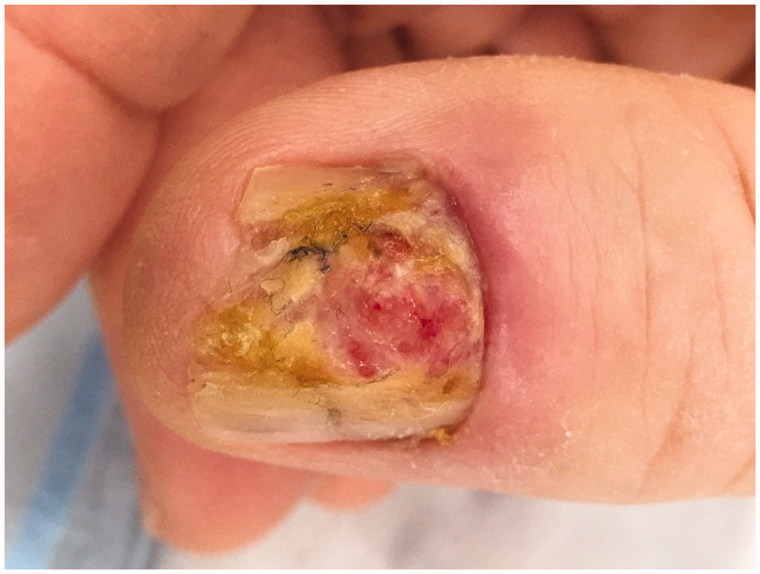
Lesion on the right thumb shown in December of 2016 prior to surgical treatment.

A 56-year-old male was referred to the plastic surgery clinic with a four-year history of a skin lesion on the dorsum of his right thumb (Figure 1). The lesion was initially diagnosed by a dermatologist as a porocarcinoma and a partial amputation of the thumb was then performed by a community plastic surgeon in December of 2016. Unfortunately, the lesion re-appeared on the distal aspect of the right thumb stump in August of 2017. The patient had no significant past medical history and was not on any medications.

**Figure 2.**
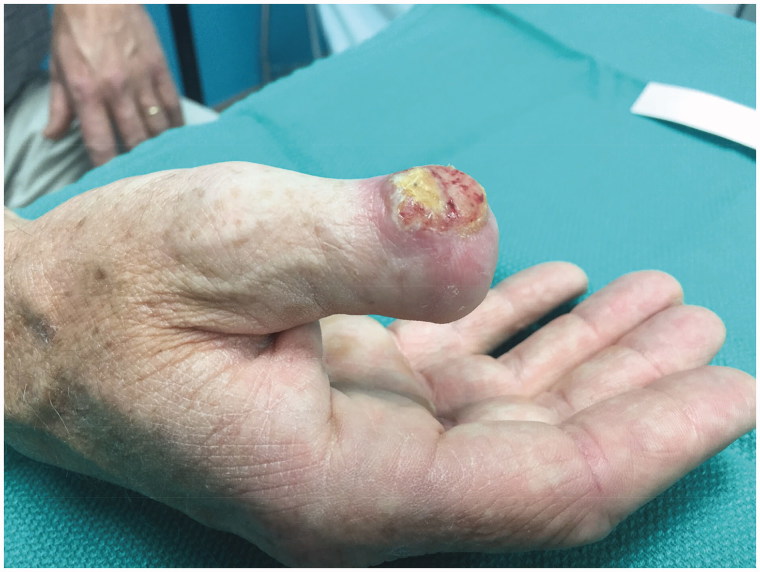
Initial patient presentation to the plastic surgery clinic in August of 2017 post initial surgery demonstrating the recurrence of the lesion on the dorsum of the right thumb interphalageal joint.

On physical examination the patient had a 1.8cm by 1.3cm well circumcised indurated lesion over the dorsum of his right thumb stump (Figure 2). The mass was firm, non-tender, and mobile over the deep structures. The patient had no palpable lymphadenopathy. The patient was then sent for a metastatic workup, which involved a computer tomography (CT) scan of the chest, abdomen, pelvis, and a bone scan. The CT scan indicated a positive enlarged lymph node in his ipsilateral axilla that was presumed to represent a metastasis.

The patient subsequently underwent a surgical excision in August of 2017 of the eccrine porocarcinoma of the thumb with an amputation at the level of the distal interphalangeal joint, and fine needle aspiration of the ipsilateral enlarged axillary node. A wide margin of 2.0 cm was used to ensure margins were clear of the tumor on the thumb. Histologic examination of the thumb specimen showed persistent ulcerated invasive eccrine porocarcinoma that involved the epidermis, dermis and extended into the subcutis with no involvement of bone. There was extensive lymphatic involvement that extended beyond the breadth of the main tumor and closely approached the peripheral margin of resection. Histologic examination of the right axillary node was positive for malignant cells that were consistent with poorly differentiated porocarcinoma and highly suspicious for metastasis. Axillary lymphadenectomy was subsequently performed and histological examination revealed metastatic involvement in 2 out of the 42 excised lymph nodes. CT examination of the rest of the body at the time of axillary resection was unremarkable.

**Figure 3.**
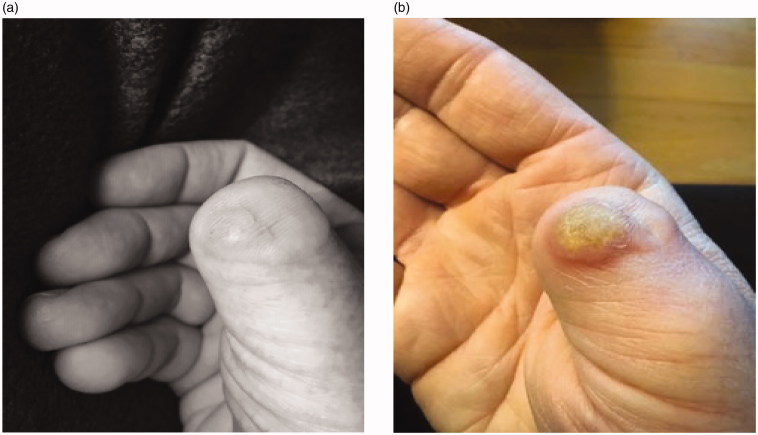
(a,b) Images taken two weeks apart showing aggressive re-occurrence of the lesion after the second surgery in April of 2018.

**Figure 4.**
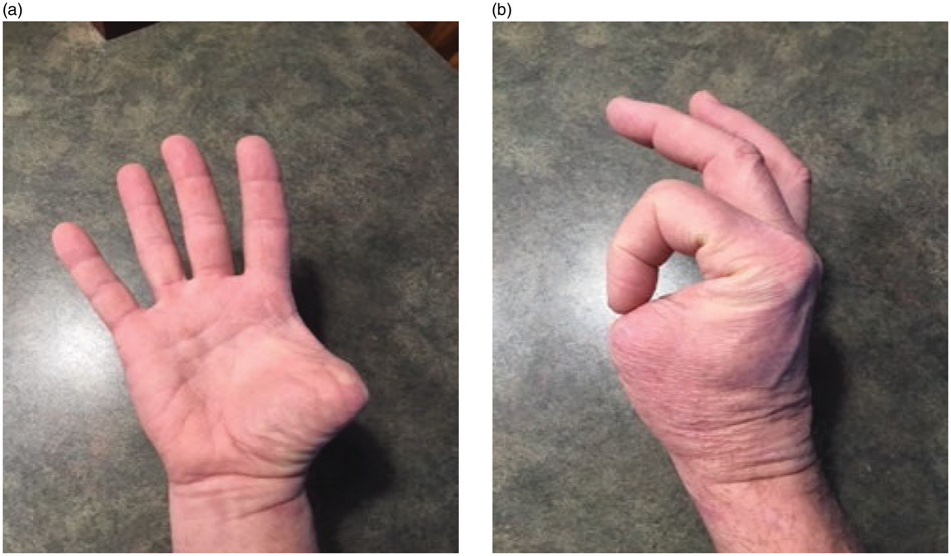
(a,b) Images taken after the third surgery in 2018 showing final functional ability.

Follow up initially was unremarkable and the wound healed well. However, four months after the operation the patient had a CT scan demonstrating a seroma in the axilla that required drainage. Seven months after surgery, a suspicious lesion was identified in the scar of the right amputated thumb (Figures 3a,b). Subsequently, the new lesion was excised with clear margins after two operations and histologic examination was positive for recurrent porocarcinoma. During the second excision the plastic surgery team completed a transfer of the flexor pollicis longus tendon to the distal bone stump to help maintain some of the adduction strength of the thumb (Figures 4 a,b). In May of 2018 the patient presented with new subcutaneous lesions. Biopsies of the right chest wall, right anterior axillary line, and right radial wrist revealed metastatic porocarcinoma. The patient received radiation therapy to the right axillary bed. Subsequent discussions with medical and radiation oncology revealed the progressing difficulty of the situation- as metastatic porocarcinoma is so rare, there are few studies investigating treatment protocols. The conversation initially shifted from curative intent to improving quality of life, however after treatments with paclitaxel (175 mg/m^2^), carboplatin (area under the curve = 5), and intralesional interleukin 2 (IL-2) injections the metastases responded with near complete disappearance of the cutaneous lesions. After one year of follow-up the patient was still responding well to this maintenance treatment.

## Discussion

There are two types of sweat glands: apocrine and eccrine. Apocrine sweat glands are typically only found in certain locations of the body like the axilla, areolas of the breast, ear canal, eyelids, perianal region, and certain parts of the genitalia [4]. The remainder of the body is covered by eccrine sweat glands, which are higher in density on the soles of the feet and palms of the hand [5]. Eccrine glands on the palms help to increase grip [5]. The gland itself is composed of an intraepidermal spiral duct, a dermal duct with a straight and coiled portion, and a secretory tubule extending through the epidermis [6].

Malignant eccrine porocarcinomas are rare, with fewer than 250 cases reported in the literature [7,8]. A review of 31 cases indicated that 65% of cases occurred on the lower extremities with the rest distributed to the head and trunk [7]. The mean age of patients was 68 years (range 42-90 years) and the distribution between genders was essentially equal. Interestingly, only four patients (12.9%) had metastases [7]. Histologic patterns were similar between specimens with most having infiltrating lymphatic vessels [5,7,9]. Histological examination of eccrine porocarcinomas typically shows tumor epithelium with large cells with irregular and hyperchromatic nuclei showing cytoplasm containing variable amounts of glycogen granules [3,10,11].The differential diagnosis for malignance eccrine porocarcinoma is broad and includes basal and squamous cell carcinomas, seborrheic keratosis, superficial spreading melanoma, fibromas, intraepidermal poroma, pyogenic granuloma, Paget’s disease, and Bowen’s disease [1,3,7].

Eccrine porocarcinomas can appear as a nodular or verrucous, dome-shaped or plaque like, erosive or infiltrative growth, and are typically ulcerated [3]. Lesions are usually red, however can be flesh colored or dark brown [3,9]. In terms of size, eccrine porocarcinomas vary considerably as case reports have shown lesions as small as 0.5cm to as large as 10cm in diameter [3,9]. Due to the variability in appearance and similarity to other common lesions like basal cell carcinomas, identification of eccrine porocarcinomas can be problematic.

The prognosis of eccrine porocarcinomas depends considerably upon the ability to metastasize. Based upon case series, approximately 20% recur at the primary site within a year of excision and 20% will metastasize to regional lymph nodes [7,10,12]. Of those cancers that metastasize, mortality rate approaches 50% although samples vary greatly [12]. Sites of metastasis are typically to the lymph nodes, however there have been instances of metastasis to the lung, long bones, breast, liver, mediastinum, peritoneum, and bladder [13–15].

To our knowledge, this is one of the first reported cases of malignant eccrine porocarcinoma of the thumb in the plastic surgery literature. Studies have shown that eccrine porocarcinomas can occasionally occur on the hand, however only a single previous case report has shown occurrence on the thumb [4,8,10,11]. Management of any eccrine porocarcinomas is principally wide excision of the lesion, with limited use of radiation or chemotherapy [16,17]. While there have been no studies formally assessing the depth and width of margin that should be excised, extrapolation from studies investigating similar tumor types shows that excision to the fascial layer may be warranted [16]. As with any large excision of the hand, tissue coverage can be an issue that may require complex reconstructive options. Close follow up to monitor for recurrence or metastasis is indicated. There may be a role for sentinel lymph node biopsy in clinically node-negative patients.

Eccrine porocarcinomas are rare and treatment planning can be challenging. In our patient, initial surgical excision did not eradicate the disease and even after multiple, more radical revisions the patient developed metastases to multiple distant sites. This patient fortunately maintained meaningful hand function despite thumb amputation, and has responded well to management of his metastatic disease with adjuvant treatments of chemotherapy and radiation therapy. Based on this experience, we would advocate for aggressive resection with wide margins to attempt to achieve local control this disease at initial surgery and hopefully prevent metastasis.
